# Cutaneous Microbial Community Variation across Populations of Eastern Hellbenders (*Cryptobranchus alleganiensis alleganiensis*)

**DOI:** 10.3389/fmicb.2017.01379

**Published:** 2017-07-21

**Authors:** Obed Hernández-Gómez, Jason T. Hoverman, Rod N. Williams

**Affiliations:** Department of Forestry and Natural Resources, Purdue University, West Lafayette IN, United States

**Keywords:** community ecology, host-associated bacteria, metacommunity, amphibian, conservation, range-wide

## Abstract

Multicellular hosts maintain complex associations with microbial communities. While microbial communities often serve important functional roles for their hosts, our understanding of the local and regional processes that structure these communities remains limited. Metacommunity analyses provide a promising tool for investigating mechanisms shaping microbiome heterogeneity, which is essential for predicting functional variation between hosts. Using a metacommunity framework, we examined heterogeneity in the skin microbiome of the eastern hellbender (*Cryptobranchus alleganiensis alleganiensis*). Hellbenders are broadly distributed throughout river systems in the eastern United States, but are present in specific environmental locations throughout their range. The large range of the species and history of population fragmentation suggest that local and regional processes contribute to the distribution of cutaneous symbiont diversity. Therefore, we characterized the skin and environmental bacterial communities at eight rivers throughout the range of the species. We observed variation among hellbender populations in skin microbial community diversity and proportion of shared operational taxonomic units (OTUs) between animal and river water communities. Among populations sampled, we noted significant clumped OTU turnover (i.e., Clementsian structure) resulting in unique cutaneous communities. In addition, we observed a significant positive correlation between skin community divergence and hellbender population genetic divergence. Host-population skin community dissimilarity did not correlate strongly with distance between sampling locations, indicating a weak spatial effect on the distribution of symbionts. These results suggest that species sorting mechanisms (i.e., local processes) structure local skin microbial communities in hellbenders. The variation in skin community composition observed among host populations foreshadows a similar pattern in important functional characteristics (e.g., resistance to dysbiosis). Future work should focus on investigating forces shaping microbiome structure in eastern hellbenders, examining functional variation among populations, and evaluating effectiveness of microbiome management recommendations.

## Introduction

In animal and plant systems, symbiont microbes provide important functional services to host physiological processes including immune system activation, metabolic regulation, energy uptake, tissue differentiation, and pathogen defense (Turnbaugh et al., 2007; Kaplan et al., 2011; Costello et al., 2012; Grice and Segre, 2012). Importantly, functionality of the host’s symbiont community can correlate with the overall diversity of the microbiome (Chang et al., 2008) or presence of key symbionts (Woodhams et al., 2007). For example, variation in the gut microbiome of mammals correlates with differences observed in digestive function between host species (Muegge et al., 2011; Phillips et al., 2012). In addition, *Actinobacteria* serve as keystone taxa in the gut of humans due to their high degree of ecological connectedness with other members of the microbiota (Trosvik and de Muinck, 2015). Given the associations between microbiota and host phenotype, understanding mechanisms shaping microbiome heterogeneity is essential to predict functional variation among hosts. While local processes (e.g., host selection) are known to influence microbial community assembly among individuals (Lindstrom and Langenheder, 2012), the contributions of regional processes (e.g., between host populations) to symbiont community structure are frequently overlooked (Mihaljevic, 2012). To provide a more comprehensive understanding of the microbiome’s significance for hosts, knowing the relative importance of local and regional processes in driving microbiome heterogeneity is key.

The amphibian skin microbiome plays an important role in resistance against pathogens (Woodhams et al., 2007). For instance, host-level variation in microbial community composition is associated with differences in immunity to the fungal pathogen *Batrachochytrium dendrobatidis* (Lam et al., 2010; Becker et al., 2015). Consequently, recent studies have focused on characterizing compositional variation in the skin microbiome between amphibian species and populations, and the local processes that shape heterogeneity in the skin microbiome. For example, antimicrobial peptides on the amphibian skin likely act as a selective filter on the microbial community (Rollins-Smith et al., 2006). Furthermore, environmental characteristics (e.g., local reservoirs, habitat quality) are correlated with variability in skin community composition among populations of the same species (Kueneman et al., 2014; Loudon et al., 2014). Thus, it is likely that throughout the range of a host species there are important alternative evolutionary associations between symbionts and hosts (Zilber-Rosenberg and Rosenberg, 2008).

Evaluating changes in host–symbiont associations across spatial scales may help inform current conservation approaches in amphibians (Jiménez and Sommer, 2017). For example, host-associated microbial communities can provide guidance in planning translocations and captive-population management. Amphibian conservation programs benefit from captive propagation and translocation strategies as a method to combat declines due to habitat loss, pollution, and emerging infectious diseases (Gagliardo et al., 2008; Zeisset and Beebee, 2013). However, divergence in microbial community composition between source and supplemented populations can negate the intended effects of translocations (Redford et al., 2012; Bahrndorff et al., 2016). Symbionts derived from captive populations can introduce pathogenic or antibiotic-resistant traits to naïve wild communities (Woodford and Rossiter, 1994). In addition, environmental variation can induce changes in the incidence or abundance of microbial symbionts (dysbiosis) that negatively impact host health (Lokmer and Wegner, 2015). To this end, amphibian management and conservation strategies may benefit from: (1) quantifying the natural distribution of bacterial symbionts across different geographic regions; and (2) establishing the relative importance of dispersal and local environmental selection in the assembly of skin microbial communities (Jiménez and Sommer, 2017).

Metacommunity theory can predict microbial community responses to eco-evolutionary processes shaping diversity at local (e.g., among individuals) and regional scales (e.g., among populations; Leibold et al., 2004). Under metacommunity theory, amphibian hosts correspond to patches of suitable habitat connected through transmission or dispersal (Mihaljevic, 2012; Christian et al., 2015). Host characteristics (e.g., immunity) and environmental variables can create a gradient of suitable habitat for microbial symbionts. Along this gradient, variation in symbiont dispersal and physiological constraints/adaptive trade-offs can result in varying patterns of taxonomic turnover (i.e., species replacements) among patches. In addition, factors that influence host movement can also bestow barriers to the dispersal of microbes throughout the host metapopulation (Mihaljevic, 2012). Quantifying skin symbiont turnover can be useful to characterize barriers to symbiont dispersal and predict important functional associations between host and symbionts across spatial scales.

Eastern hellbenders (*Cryptobranchus alleganiensis alleganiensis*) serve as an ideal model to investigate community structure and distribution patterns of cutaneous microbes. This species has exhibited population declines and low levels of recruitment within the last 30 years (Wheeler et al., 2003). Hellbenders are habitat specialists restricted to lotic habitats and have a large range throughout the eastern United States (Nickerson and Mays, 1973). Their dispersal is approximately linear and influenced by flood events (Humphries, 2005) and/or changes in river architecture (Quinn et al., 2013). Recent population genetic assessments using microsatellite markers found two genetic demes (Routman et al., 1994; Sabatino and Routman, 2009; Tonione et al., 2011; Unger et al., 2013) restricted to the major watersheds where these salamanders are found (i.e., the Ohio River Drainage and the Tennessee River Drainage; Unger et al., 2013). This genetic information is currently implemented in the execution of conservation management actions (e.g., translocations). However, the expansive range of the species could lead to variance in environmental conditions among populations (e.g., water quality and temperature). Environmental effects combined with limited host dispersal can lead to variation among the skin microbial communities of individuals within the hellbender metapopulation. Thus, characterizing skin microbiome turnover among individual communities throughout the range of the eastern hellbender may benefit current conservation approaches by providing an additional guide for translocations.

We implemented culture-independent microbiome characterization methods to identify cutaneous bacterial communities on the skin of eastern hellbenders and river water. We obtained skin and water samples from hellbender populations in different rivers throughout the range of the subspecies. We then tested for differences in skin community diversity and the proportion of shared microbes between water and skin communities among populations. Due to the large range of the species, we predicted that bacteria richness/diversity and the proportion of input from environmental reservoirs would differ among populations. We also evaluated metacommunity structure of hellbender skin communities to quantify species turnover among individuals. While we predicted positive turnover among individuals, we expected more prominent turnover among populations due to variation in environmental characteristics (e.g., elevation, latitude, land use). In addition, we assessed the influence of dispersal on local community structure. We predicted a positive correlation between geographic distance and community dissimilarity in response to limits to hellbender dispersal. Finally, we tested for differences in community composition between the two genetic demes and between hellbenders from each population (i.e., river). We expected to observe differentiation between both genetic demes and among each locality (species sorting paradigm) given that host dispersal and environmental heterogeneity can influence skin community assembly in amphibians.

## Materials and Methods

### Field Methods

**FIGURE 1:**
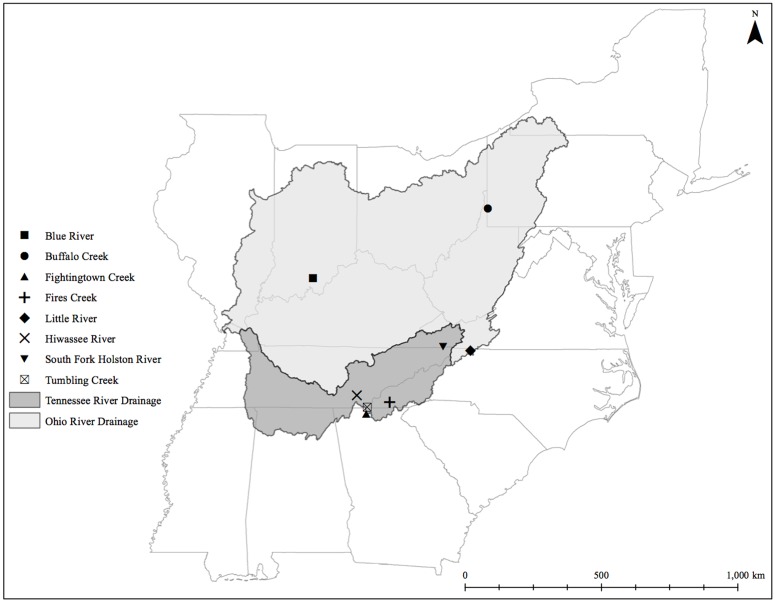
Eastern hellbender cutaneous microbiome sampling sites across the subspecies range.

We sampled eastern hellbenders between June 18 and July 31, 2014. Our sampling occurred throughout five states (**Figure 1**; Indiana, West Virginia, North Carolina, Tennessee, and Georgia), making sure to include sites across the range for each of the two genetic demes described in Unger et al. (2013). We handled hellbenders following an approved protocol by the Purdue University Animal Care and Use Committee (PACUC protocol # 14060011094). We captured and sampled the microbiome of each individual following the protocol of Hernández-Gómez et al. (2017). We returned all hellbenders to their original location of capture within the river after swabbing was complete. From each population, we also collected two liters of water in sterilized Nalgene bottles 1–10 m upstream from where sampling began. We stored the bottles on ice for up to 2 h until filtration occurred in an aseptic environment. We chose to filter the water on a Whatman #1 11 μm filter paper (GE Healthcare, Chicago, IL, United States) due to the high siltation in several water samples. These filters helped to reduce clogging and filtering times. Given the size of the filters, we expected to have captured bacteria attached to particulate matter only, and not the whole planktonic community. We stored the filters in 15-mL centrifuge tubes, and placed them in liquid nitrogen before moving them to a -80°C freezer until DNA isolation occurred.

### Environmental Data Collection

For each population sampled, we recorded GPS coordinates at the position where sampling began, and estimated linear distance between sampled sites *post hoc* using Google Earth (Google, Mountain View, CA, United States). We obtained land-use, latitudinal, and elevational data from publically available GIS databases. We chose to include these layers as they have been previously associated with stream microorganism biogeography (Lear et al., 2013). We used the USGS National Watershed Boundary Dataset to identify the HUC 12-level boundaries at each river sampled in ArcGIS 10.2 (Esri, Redlands, CA, United States). We applied these boundaries to characterize the percentage of land covered in forest using the National Land Cover Data from 2006 (Fry et al., 2011). Because we did not obtain sampling points for each hellbender, we randomly assigned geographic coordinates for each individual along the estimated sampling length within its river. At each pseudo-coordinate, we obtained elevation data from the USGS Elevation Point Query Service.

### Laboratory Methods

We isolated DNA from skin swabs using the PowerSoil DNA Isolation Kit (MoBio Laboratories, Inc., Carlsbad, CA, United States) following the protocol described in Hernández-Gómez et al. (2017). We processed water filters using the PowerWater DNA Isolation Kit (MoBio Laboratories, Inc., Carlsbad, CA, United States) following manufacturer’s instructions. We prepared the sequencing library using two sequential PCRs. The first PCR consisted of amplification of the 16S rRNA V2 region using primer pair 27F/338R (Fierer et al., 2008) with the attachment of connector sequences (Hernández-Gómez et al., 2017). We ran each sample in triplicate, and each reaction consisted of 5 μL of template DNA, 12.5 μL of MyTaq Master Mix (Bioline, Tauton, MA, United States), 1 μL of 10 mM forward and reverse primers, and 6.5 μL of water (MoBio Laboratories, Inc., Carlsbad, CA, United States) for a total of 25 μL per reaction. PCR conditions consisted of 95°C for 2 min, 30 cycles of 94°C for 45 s, 50°C for 60 s, and 72°C for 90 s, followed by 72°C for 10 min. We pooled triplicates and cleaned the PCR products using the UltraClean PCR Clean-up kit (MoBio Laboratories, Inc., Carlsbad, CA, United States). The second PCR was performed to add-on dual-index barcodes connected to Illumina sequencing adaptors (Hernández-Gómez et al., 2017) to the ends of amplicons. The PCR consisted of 5 μL of clean amplicons, 12.5 μL of MyTaq Master Mix, 1 μL of forward and reverse barcode primers, and 6.5 μL of water for a total of 25 μL reactions. PCR conditions consisted of 95°C for 2 min, 5 cycles of 94°C for 45 s, 65°C for 60 s, and 72°C for 90 s, followed by 72°C for 10 min. We quantified the PCR products using a Qubit Fluorometer (Invitrogen, Corp, Carlsbad, CA, United States), and pooled the samples in equimolar amounts to be cleaned using the UltraClean PCR Clean-Up kit. The cleaned sample pool was sequenced on a MiSeq machine (Illumina, Inc., San Diego, CA, United States) utilizing the Reagent Kit V2 to produce 250 bp paired end reads.

### Sequence Analysis

We processed raw reads using Trimmomatic (Bolger et al., 2014) to remove adapter sequences, bases below threshold quality of phred-20 from both ends of reads, and any resulting reads under 30 bp. We paired reads that passed initial quality control using PANDAseq (Masella et al., 2012). Only reads that paired successfully were utilized in subsequent analysis. We used a custom Python script to remove quality scores from reads and re-name the reads with a name compatible with our chosen analysis pipeline. We processed the resulting read file using the Quantitative Insights Into Microbial Ecology version 1.8.0 (QIIME) pipeline (Caporaso et al., 2010b). We clustered reads at the standard 97% similarity using the open-reference protocol (Rideout et al., 2004) and the Greengenes 13_5 reference database (DeSantis et al., 2006). Reads that failed to cluster using the open-reference algorithm were clustered into *de novo* operational taxonomic units (OTUs) with UCLUST (Edgar, 2010). OTUs that were clustered using the Greengenes database retained the matched taxonomy, while *de novo* OTUs were assigned taxonomy using the RDP Classifier (Wang et al., 2007) at 80% confidence. We aligned representative sequences to the pre-aligned Greengenes reference using PyNAST (Caporaso et al., 2010a) and the alignment was used to produce a phylogenetic tree through FastTree (Price et al., 2010).

To avoid including OTUs generated by sequencing error (e.g., chimeras and base miscalls) we removed OTUs that were represented by fewer than 0.005% of the total read count (Bokulich et al., 2013). We also rarified sequencing depth to 8,800 reads per sample to standardize our samples. We calculated the core microbiome (defined as the set of detectable OTUs present in at least 80% of all samples) to derive the ubiquitous OTUs across the range of the eastern hellbender.

### Data Analysis

We conducted a series of analyses to explore the skin microbiota of hellbenders. In brief, we evaluated differences in richness and diversity between the cutaneous microbial communities of hellbenders from different populations. In addition, we implemented metacommunity analysis to assess patterns in bacterial OTU distribution across the eastern hellbender metapopulation. Lastly, we assessed differences in community composition between the two genetic demes of eastern hellbenders and among the sampled populations. We performed all statistical analyses in R version 3.2.0 unless otherwise noted.

#### Richness and Diversity Comparison

We assessed differences in community richness/diversity between the two genetic demes and among populations, and compared the proportion of shared OTUs between skin and water samples at each population. We calculated richness (observed OTUs) and Shannon Diversity Index values within each sample from the abundance-based OTU table in QIIME. We used richness or Shannon Diversity Index values for each hellbender as dependent variables, genetic deme assignment as a fixed factor, and population as a random factor in negative binomial generalized linear mixed models (richness) or linear mixed models (Shannon Diversity Index). In addition, we assessed pairwise differences in richness and Shannon Diversity Index between all populations using Tukey’s honest significant difference (HSD) tests. We also calculated the proportion of OTUs shared between hellbenders and the corresponding river water samples. We used the proportion of shared OTUs as dependent variables, genetic deme assignment as fixed factor, and population as random factors in a generalized linear mixed model. Lastly, we tested pairwise differences in proportion of shared OTUs between all populations using Tukey’s HSD tests.

#### Metacommunity Structure

We assessed the metacommunity structure as described in Leibold and Mikkelson (2002) and Presley et al. (2010) using the package metacom in R (Dallas, 2014). From the QIIME generated OTU table, we created an individual hellbender by OTU matrix. We used reciprocal averaging (i.e., correspondence analysis) to rank hellbenders so that those having similar OTU composition are close to each other, and OTUs were ordinated so that those having similar occurrence among the hellbenders were close to each other. OTU distribution patterns were derived by calculating the three elements of metacommunity structure (EMS): coherence, turnover, and boundary clumping. *Coherence* is a measure of the number of embedded species absences (gaps within a species range) within an ordinated community matrix. Negative coherence (more embedded absences within the matrix than expected by random chance) indicates a “checkerboard” metacommunity structure. Positive coherence indicates the response of species to a gradient of environmental variation, and denotes the need to evaluate species turnover and boundary clumping to identify structure (Leibold and Mikkelson, 2002). *Species turnover* represents the number of times that one species replaces another between sites. Negative turnover (denoted as lower replacements than expected by chance) is associated with a nested structure while positive turnover (larger replacements than expected by chance) is associated with Gleasonian or Clementsian structures (Leibold and Mikkelson, 2002). *Boundary clumping* is evaluated through the calculation of Morisita’s index; a value greater than one corresponds with a Clementsian structure (i.e., clumped range boundaries), a value less than one corresponds with an even spaced structure (hyperdisplaced range boundaries), and a value equal to 1 corresponds with a Gleasonian structure (randomly distributed boundaries). We implemented the default ‘r1’ fixed-proportional null model to calculate the significance of the EMS indices calculated. The fixed-proportional model maintains the OTU richness within each locality while filling OTU ranges based on their marginal probabilities. Because we were working with a very large dataset, we restricted permutations to 99 and allowed null matrices to have empty rows and columns as recommended by Dallas (2014) to reduce computation time.

We chose to include all OTUs in the analysis even though a high proportion of OTUs in our dataset were rare (∼77.5% of OTUs had a total sequencing depth of less than 100 sequences). While metacommunity analysis was developed for macro-communities with fewer species, it has been effectively implemented to characterize the metacommunity structure of bacterial communities before (Heino et al., 2015). As such, we followed the methodology of Heino et al. (2015) in our approach to characterize the metacommunity of hellbender microbial communities. We also performed a trial analysis to compare the structure of the complete dataset and the community data without rare species. Because we did not find a difference in metacommunity structure between both approaches, we only present analysis/results on the complete dataset. We performed the metacommunity analysis three times. For the first analysis, we used an OTU-by-hellbender incidence matrix that included all skin samples throughout the range. To evaluate the effect of environmental variables on hellbender ordination, we performed Pearson correlation tests between sample ordination scores and HUC 12-level percent forest cover, elevation, and latitude. Upon performing the first analysis, we noted a pattern of clumped OTU turnover between two groups of samples (i.e., compartments). These compartments formed non-random groupings of hellbender populations; thus, we decided to evaluate the metacommunity structure of each compartment independently to evaluate species turnover as suggested in Presley et al. (2010). We split the data matrix on this boundary and performed the metacommunity structure analysis on each of the two compartments.

#### Microbial Community Structure

For all community composition comparisons, we transferred the OTU tables and corresponding Newick phylogenetic tree to R, and the package GUniFrac was used to build UniFrac distance matrices (unweighted and weighted; Lozupone et al., 2011). We performed Adonis and ANOSIM tests using the unweighted and weighted UniFrac distance matrices within the package vegan 2.2-1 (Oksanen et al., 2007) to partition the variation between groups. To assess the influence of genetic deme assignment on the microbiota of eastern hellbenders, we tested differences between skin samples assigned to each major drainage (i.e., Ohio or Tennessee). We used major watershed identity as a representative of genetic deme assignment as described in Unger et al. (2013). To evaluate the influence of population on the microbiota of eastern hellbenders across the range, we tested differences between skin samples assigned to each population. We visualized differences in community structure using unweighted and weighted UniFrac distances through a principal coordinate analysis (PCoA) generated through package phyloseq in R (McMurdie and Holmes, 2013).

To evaluate the effect of population-level genetic differentiation we conducted correlations between microbial community distances and genetic/geographic distances (Fst). To evaluate population genetic distance, we obtained microsatellite genotype data from Unger et al. (2013) for each of the populations sampled in this study. Unger et al. (2013), did not sample the Little River, NC population; thus, we obtained genotypes for the closest population in the respective drainage system (South Fork of the New River). We calculated population genetic distances (Fst) using the package hierfstat in R (Goudet, 2005). We assessed correlations between Fst and UniFrac (unweighted and weighted) using a Mantel test. Lastly, we assessed the correlation between linear distance and community dissimilarity to evaluate dispersal barriers. We uploaded the pseudo-coordinates to QGIS 2.18 (Open Source Geospatial Foundation Project)^1^, and calculated linear distances between all points. In R, we conducted a Mantel test using the pairwise spatial distance and community dissimilarity (1-Jaccard) matrices. The derived correlations were compared to correlations originated from 10,000 random permutations.

## Results

**FIGURE 2:**
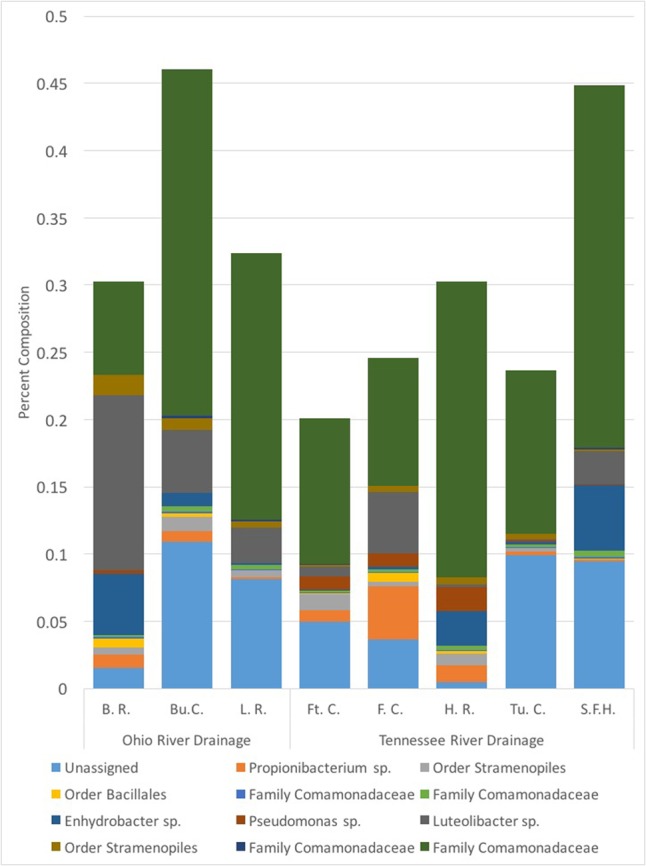
Core skin microbial operational taxonomic units (OTUs – 80% prevalence) of eastern hellbenders and the mean relative abundance of those OTUs within each locality sampled. Core microbiome across the eastern hellbender range is dominated by similar taxa. (Localities: BuC – Buffalo Creek, WV; SFH – South Fork of the Holston, VA; TuC – Tumbling Creek, TN; HR – Hiwassee River, TN; FC – Fires Creek, NC; LR – Little River, NC; BR – Blue River, IN; FtC – Fightingtown Creek, GA).

We collected samples from three populations within the Ohio River Drainage (Blue River, Indiana-BR, *n* = 5; Buffalo Creek, West Virginia-BuC, *n* = 5; and Little River, North Carolina-LR, *n* = 8) and five populations within the Tennessee River Drainage (Hiwassee River, Tennessee-HR, *n* = 5; Tumbling Creek, Tennessee-TC, *n* = 3; Fightingtown Creek, Georgia-FT, *n* = 5; South Fork of the Holston River, Virginia-SFH, *n* = 8; and Fires Creek, North Carolina-FC, *n* = 5) for a total of 43 hellbender and eight water samples (Supplementary Table S1). Amplicon sequencing resulted in more than 10 million reads with an average length of 344 base pairs. We deposited sequencing data into the NCBI Sequence Read Archive (accession numbers: SRR5479798-?SRR5479789). A total of 1,656 OTUs were identified across all adult swabs and water samples after filtration and rarefication. Hellbender skin bacterial communities were dominated by a few abundant OTUs. The core microbiome of eastern hellbenders was dominated by nine OTUs belonging to the phyla Proteobacteria, Actinobacteria, Firmicutes, Cyanobacteria, and Verrucomicrobia (**Figure 2**). OTUs with the highest abundance on the skin of eastern hellbenders were assigned to the family Comanmonadaceae (mean relative abundance: 46.5%), order Stramenopiles (9.4%), and the genus *Proprionibacterium* (8.4%).

### Richness/Diversity Comparisons

**FIGURE 3:**
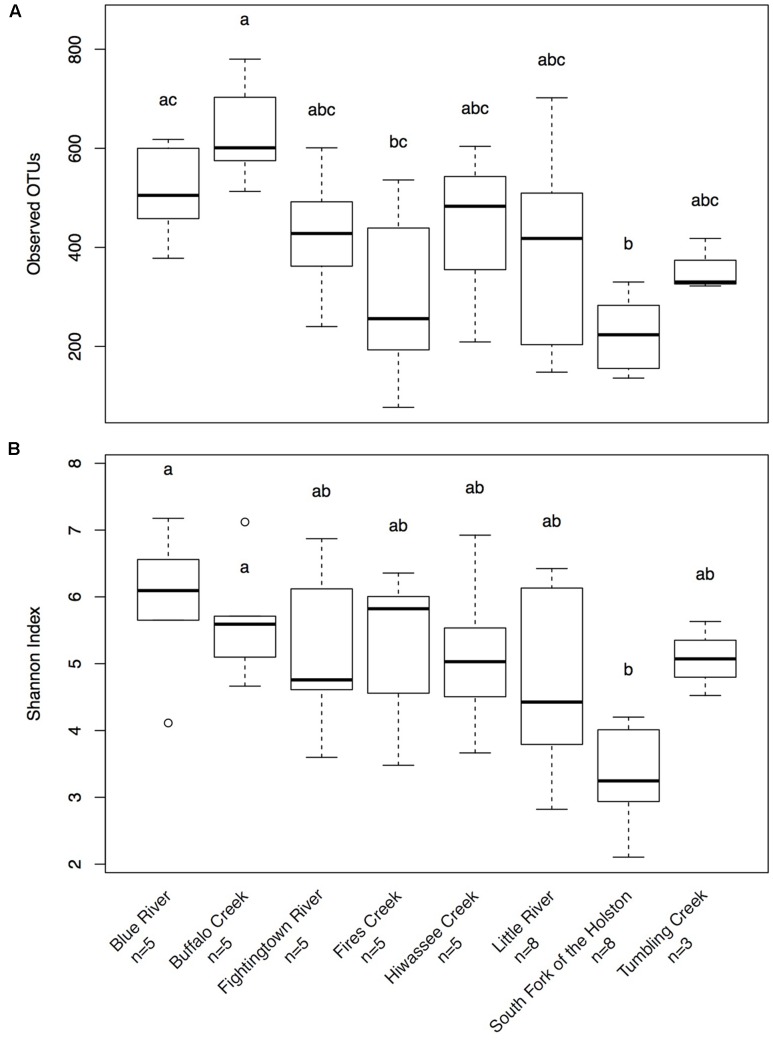
Box-plot distributions of alpha diversity values **(A)**- Observed OTUs, **(B)**- Shannon Diversity Index for cutaneous microbial communities from eastern hellbenders at different localities in the subspecies’ range. There is more variation in values of observed OTUs among populations over Shannon Diversity Index. Letters (a, b, c) indicate significant differences among hellbender populations using *post hoc* Tukey’s HSD tests. Outliers are noted by circles.

**FIGURE 4:**
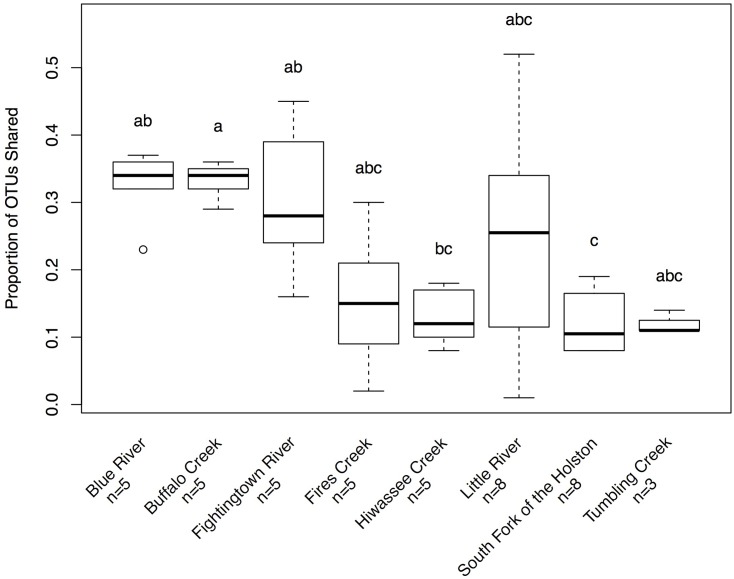
Box-plot distributions of the proportion of OTUs shared between hellbenders and river water samples at different localities in the subspecies’ range. Populations of eastern hellbenders vary in the proportion of OTUs shared between the skin and river water. Letters (a, b, c) indicate significant differences among hellbender populations using *post hoc* Tukey’s HSD tests. Outliers are noted by circles.

Hellbenders from the Ohio River Drainage possessed richer communities (observed OTUs values; mean ± SD: 491.28 ± 178.09) than hellbenders from the Tennessee River Drainage (mean ± SD: 333.77 ± 149.29; LRT = 3.75, *p* = 0.053). Shannon Diversity Index values did not differ between the two genetic demes (LRT = 1.25, *p* = 0.263). We found significant differences in skin community richness and diversity among sampling localities from *post hoc* multiple comparisons (**Figure 3**). With respect to the proportion of shared OTUs between individuals and river water, hellbenders from the Ohio River Drainage (mean ± SD: 0.29 ± 0.12) shared a higher proportion of OTUs with the river water compared to hellbenders from the Tennessee River Drainage (mean ± SD: 0.16 ± 0.10; LRT = 6.00, *p* = 0.014). Finally, we found significant differences among sampling localities in the proportion of shared OTUs between river water and the skin from *post hoc* multiple comparisons (**Figure 4**).

### Metacommunity Analysis

**FIGURE 5:**
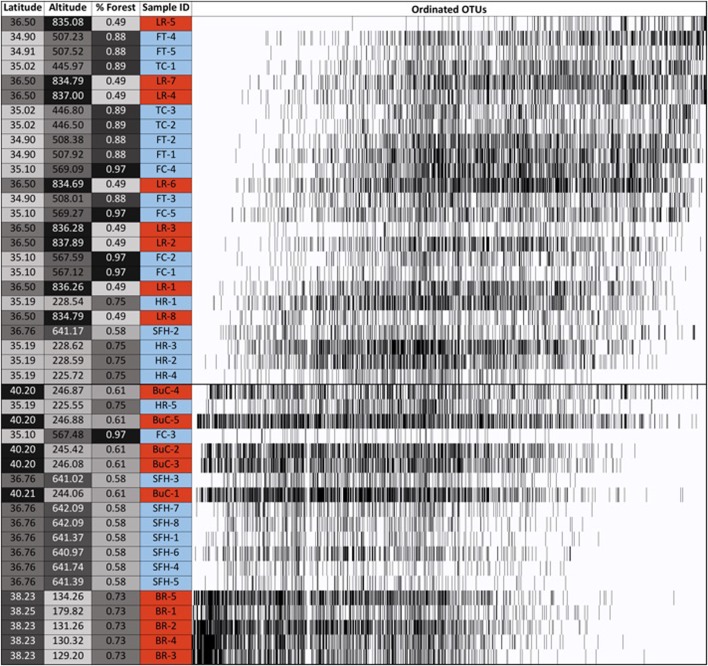
Incidence matrix for skin bacterial communities from eastern hellbenders. Skin samples and operational taxonomic units (OTUs) were ordinated using reciprocal averaging as described in Leibold and Mikkelson (2002). Solid bars indicate each OTU’s presence throughout the ordinated hellbenders. Sample labels correspond to rivers where hellbenders were sampled from: BuC – Buffalo Creek, SFH – South Fork of the Holston, TuC – Tumbling Creek, HR Hiwassee River, FC – Fires Creek, LR – Little River, BR – Blue River, and FtC – Fightingtown Creek. Sample label color corresponds with the two hellbender genetic demes (Ohio River Drainage – red, Tennessee River Drainage – blue). Latitude, altitude, and % forest cover data is presented and labeled in gray scale from lowest (light) to highest (dark) values. Metacommunity structure analysis indicated Clementsian structure corresponding with clumped OTU turnover. The horizontal line marks boundary of OTU boundaries generating two major compartments.

The fixed-proportional null model in the EMS analysis revealed that the hellbender cutaneous bacterial metacommunities displayed positive coherence (absences: 44,120; simulated mean: 52,216 ± 668.0; *p* < 0.001) and positive turnover (replacements: 6,764,076; simulated mean: 552,825.38 ± 205,075.34; *p* < 0.001). In addition, we found significant positive boundary clumping in the distribution of OTUs (Morisita’s index = 72.41, *p* < 0.001, df = 1,642). Together, these three values indicate a Clementisan pattern in skin microbial metacommunities throughout the eastern hellbender range. We noted significant correlations between hellbender sample ordination scores and elevation (*r* = 0.58, *p* < 0.001) and latitude (*r* = -0.48, *p* = 0.002). However, we did not note a significant correlation between sample ordination scores and HUC 12-level percent forest cover (*r* = 0.25, *p* = 0.106). There was a discernable clumping of OTU boundaries (**Figure 5**) that separated two major compartments of ordinated samples corresponding mostly to the Blue River, Buffalo Creek, and South Fork of the Holston River (compartment 1) and the Little River, Hiwassee River, Tumbling Creek, Fightingtown Creek, and Fires Creek (compartment 2). However, the boundary between these compartments did not align as expected between the two eastern hellbender genetic demes (**Figure 1**).

**FIGURE 6:**
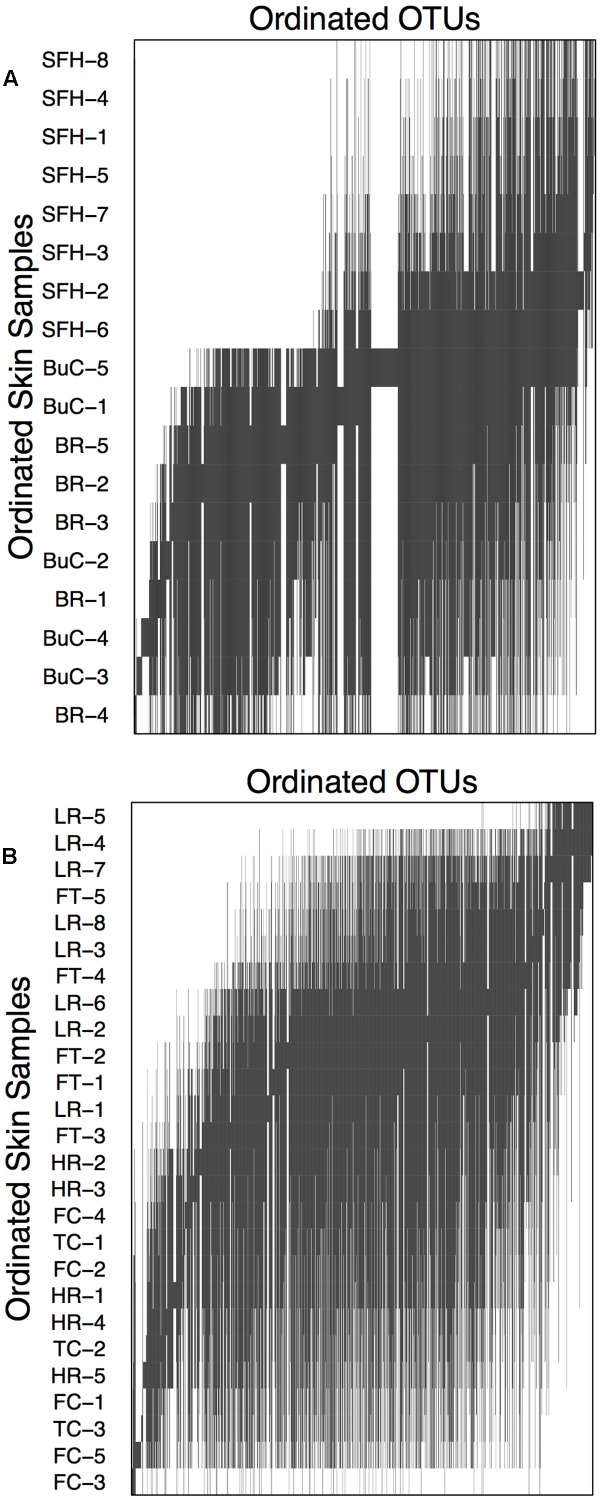
Compartment incidence matrices for skin bacterial communities from eastern hellbenders. Compartments correspond to the species distribution boundary outlined in **Figure 5**. Skin samples and OTUs were ordinated using reciprocal averaging as described in Leibold and Mikkelson (2002). Solid bars indicate each OTU’s range throughout the ordinated hellbender samples. Within both compartments, metacommunity structure corresponds with Clementsian structure. Sample labels correspond to rivers where hellbenders were sampled from BuC – Buffalo Creek, WV; SFH – South Fork of the Holston, VA; BR – Blue River, IN for compartment in **(A)** and TuC – Tumbling Creek, TN; HR Hiwassee River, TN; FC – Fires Creek, NC; LR – Little River, NC; and FtC – Fightingtown Creek, GA for compartment in **(B)**.

Within both compartments cutaneous bacterial metacommunities displayed Clementsian structure as evidenced by positive coherence (compartment 1: absences = 11,154, simulated mean = 15,127.41 ± 537.14, *p* < 0.001; compartment 2: absences = 22,558, simulated mean = 26,512.00 ± 622.17, *p* < 0.001), positive turnover (compartment 1: replacements = 1,601,524, simulated mean = 268,412.29 ± 78,876.94, *p* < 0.001; compartment 2: replacements = 3,819,214, simulated mean = 411,109 ± 78,876.94, *p* < 0.001), and significant positive boundary clumping (compartment 1: Morisita’s index = 38.59, *p* < 0.001; compartment 2: Morisita’s index = 115.22; *p* < 0.001). Within compartment 1, sample grouping is noticeable between hellbenders from the Blue River/Buffalo Creek and the South Fork of the Holston River (**Figure 6A**). Within compartment 2, grouping is noticeable between hellbenders from Fightingtown Creek/Little River and Hiwassee River/Fires Creek/Tumbling Creek (**Figure 6B**). Samples from compartment 1 were collected from sites with higher latitudes (mean ± SE; 38.13 ± 0.35 vs. 35.50 ± 0.13°), lower elevation (392.56 ± 54.88 vs. 595.33 ± 43.23 m), and lower HUC 12-level percent forest cover (62.94 ± 1.61 vs. 75.39 ± 3.75%) than samples form compartment 2.

### Microbial Community Structure

**FIGURE 7:**
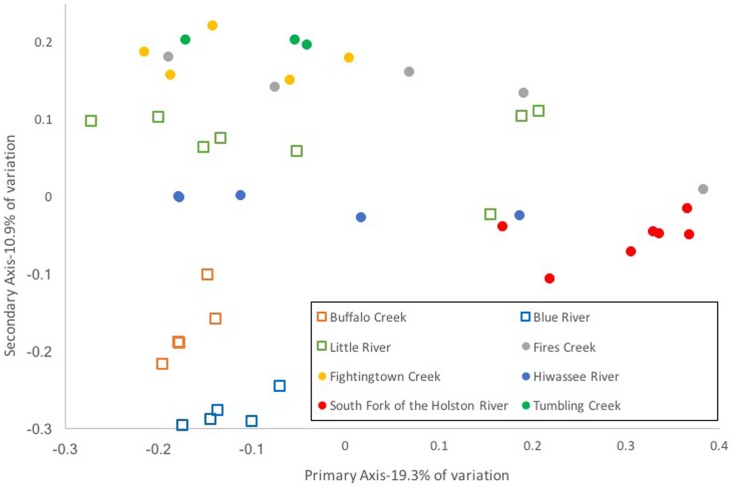
Principal coordinate analysis of un-weighted UniFrac distance matrices from hellbender skin microbial communities at eight rivers in the species range. Each point represents the skin bacterial community of an individual hellbender; shape indicates genetic deme assignment (squares -Ohio River Drainage, circles -Tennessee River Drainage) and color indicates river locality. While there is no distinct clustering of points by genetic deme, clustering by river locality is visible.

**FIGURE 8:**
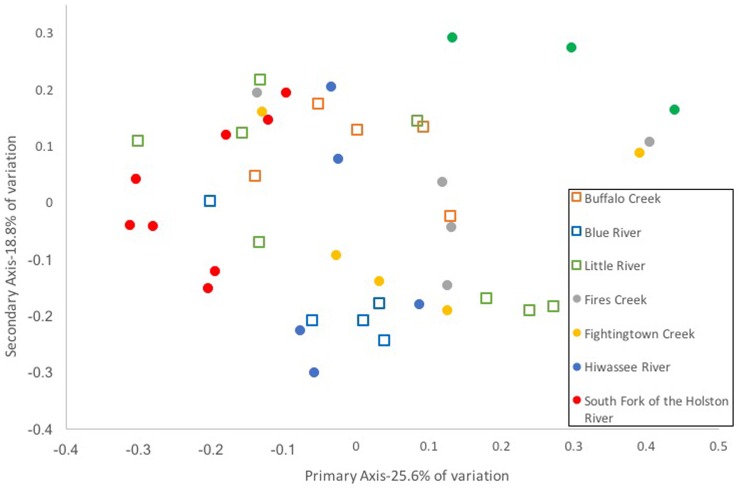
Principal coordinate analysis of weighted UniFrac distance matrices from hellbender skin microbial communities at eight rivers in the species range. Each point represents the skin bacterial community of an individual hellbender; shape indicates genetic deme assignment (squares -Ohio River Drainage, circles -Tennessee River Drainage) and color indicates river locality. While there is no distinct clustering of points by genetic deme or river locality.

Composition of the hellbender skin microbiome varied more strongly by population than by genetic deme. Community composition between the two genetic demes varied moderately when based on presence/absence of OTUs alone (unweighted UniFrac: Adonis *R* = 0.09, *p* < 0.001; ANOSIM *R* = 0.20, *p* = 0.001). However, no variation was noted when abundance of OTUs was taken into account (weighted UniFrac: Adonis *R* = 0.02, *p* = 0.285; ANOSIM *R* = 0.05, *p* = 0.023). In contrast, a strong difference among sampling localities was noted when comparing microbiome communities using unweighted (Adonis *R* = 0.42, *p* < 0.001; ANOSIM *R* = 0.56, *p* < 0.001) and weighted UniFrac distances (Adonis *R* = 0.40, *p* < 0.001; ANOSIM *R* = 0.37, *p* < 0.001). PCoA graphs display clear partitioning of points based on population over genetic sub-population for unweighted UniFrac distances (**Figure 7**) and weak grouping for weighted UniFrac distances (**Figure 8**). There were significant positive correlations between skin community dissimilarity and genetic distances (unweighted UniFrac: *r* = 0.39, *p* < 0.001; weighted UniFrac: *r* = 0.31, *p* < 0.001). There was a weak, but significant, correlation between skin community dissimilarity and distance throughout the range of the species (*r* = 0.12; *p* = 0.035).

## Discussion

We implemented culture-independent microbiome characterization and microbial community analyses to describe the eastern hellbender cutaneous microbiome across the range of the subspecies. We observed variation among populations in skin microbial community richness, diversity, and the proportion of shared OTUs between animal and environmental samples. The metacommunity analysis revealed Clementsian structure, which suggests clumped species turnover among groups of samples. We observed compartmentalization within the hellbender skin microbiome metacommunity that aligned with changes in latitude, elevation, and HUC 12-level percent forest cover. The effect of environmental heterogeneity on community assembly in the skin of hellbenders is further evident given that OTU turnover correlated weakly with linear distance. Finally, OTU turnover among populations resulted in phylogenetically distinct communities. Together, the results suggest there is a large amount of regional-level variation in the skin bacterial communities of eastern hellbenders. Large-scale variation is formed by symbiont turnover among hellbender populations in response to differences in local processes.

Eastern hellbenders occupy a large range that encompasses broad variation in environmental factors (e.g., stream order, habitat quality, temperature; Hanson et al., 2012); therefore, it is likely that OTU turnover among populations occurred in response to environmental differences. In our metacommunity analysis, we were able to detect significant bacterial OTU turnover with changes in latitude and elevation. While we lack real-time environmental data (e.g., salinity, pH, temperature) to evaluate OTU turnover against stream characteristics, we can infer that climatic variability may have an influence on the skin microbiome of hellbenders. In addition, we only evaluated overlap between particle-attached communities in the river water and the skin of the salamanders ignoring other possible environmental reservoirs (e.g., free-living, sediment, cover-rocks, alternative hosts). Furthermore, we did not test for *Batrachochytrium dendrobatidis* or other integumentary pathogens in this study. While evaluating the presence of specific pathogens was not within the scope of our study, the presence of these pathogens can alter the microbial communities of amphibians (Jani and Briggs, 2014). Identifying environmental variables, reservoirs, and biotic factors that influence the assembly of symbiont communities is pertinent to pinpoint factors that contribute to functional variation among populations or individuals. While we lacked an environmental component, we still implemented community structure patterns to draw conservative inferences regarding possible functional variation within the microbiome of this salamander.

### Variation in Skin Community Richness/Diversity

We observed higher skin bacterial community richness in hellbenders from the Ohio River Drainage compared to hellbenders from the Tennessee River Drainage. In addition, bacterial communities varied in richness among hellbender populations. Differences in community richness likely result from different environmental conditions (e.g., temperature, stream order, habitat quality) between populations. Despite differences in richness, we did not observe significant variation in community diversity (i.e., Shannon Diversity Index) between the two genetic demes or among populations. An absence of variation in community diversity likely resulted from similar patterns of OTU abundances (skewed toward a few abundant OTUs) throughout the range.

Variation in community richness among populations can translate into differences in community functional diversity. Within macro- and microbial systems, it is common to observe a relationship between biodiversity and ecosystem functionality (Balvanera et al., 2006). More importantly, symbiont diversity may correlate with the ability of microbial communities to resist or recover from disturbances (i.e., dysbiosis) generated from environmental pressures or pathogen invasion (Girvan et al., 2005; Lozupone et al., 2012). Diversity is important to community stability as it increases the probability that functionally important bacteria are present in the environment, or can enhance synergistic effects (Loreau and Hector, 2001). In humans, loss of gut microbiome diversity due to recurrent antibiotic use is associated with recurrent *Clostridium difficile* infections (Chang et al., 2008). Within amphibians, skin community diversity is linked with the ability to suppress *Batrachochytrium dendrobatidis* growth (Rebollar et al., 2016; Piovia-Scott et al., 2017). While much of the research on amphibian microbial immunity is concentrated on the contributions by individual bacterial species, recent investigations show that defense might depend on overall community diversity (Lam et al., 2010; Jani and Briggs, 2014; Piovia-Scott et al., 2017). Therefore, within the range of the eastern hellbender, populations possessing richer skin communities may possess increased ability to resist dysbiosis induced by disease or environmental changes (e.g., translocation, disease, pollution) than those with low richness.

### Clumped Skin Bacterial Lineage Turnover

In addition to diversity differences, bacterial turnover in skin communities among our hellbender samples may result in functional heterogeneity. Within amphibians, OTU turnover among populations of the same host species is not a novel finding (Lam et al., 2010; Kueneman et al., 2014; Rebollar et al., 2016). However, this study is the first to implement metacommunity structure analysis to quantify the magnitude of species turnover among individuals and populations. In our observations, clumped species turnover (i.e., Clementsian structure) resulted in unique communities among two major compartments (compartment 1: Blue River, Buffalo Creek, South Fork of the Holston River; compartment 2: Fightingtown Creek, Fires Creek, Hiwassee River, Little River, Tumbling Creek; **Figure 5**). This structure indicates similarities among groups of OTUs in their adaptive trade-offs or tolerances to local environmental factors (Clements, 1916; Presley et al., 2010). In addition, clumped turnover of bacterial skin symbionts to environmental pressures could indicate replacement of functionally important traits. For example, patterns of symbiont turnover are associated with loss/gain of anti-pathogen metabolite producing bacteria on the skin of amphibians (Lam et al., 2010). The complete replacement of symbionts among the observed compartments in the metacommunity could translate into major differences in community functionality. Integrating ‘-omic’ approaches to characterize functionality of the skin communities may help in quantifying functional redundancy in the regional pool of hellbender skin symbionts. For example, metagenomics was successful in describing functional redundancy among gut microbiota in humans despite disparity in community composition (Ferrer et al., 2013). Belden et al. (2015) implemented high-performance liquid chromatography-mass spectrometry to characterize skin microbe metabolites, and identified a lack of differentiation among host species despite community dissimilarity. Thus, incorporating functional characterization in future assessments of hellbender skin will be useful to evaluate how OTU turnover influences important functional traits, such as pathogen defense or community resistance to anthropogenic activities (Jiménez and Sommer, 2017).

### Skin Community Compositional Differences

Implementing a phylogenetic based dissimilarity index to characterize beta diversity among hellbender skin communities allowed us to assess bacterial lineage turnover. We expected to observe segregation between samples of each genetic deme, given that historic host movement may have monopolized host bacterial lineages within each genetic deme (Mihaljevic, 2012). Eastern hellbender communities throughout the range were dominated by a few taxa. As such, we did not observe a strong pattern of differentiation from weighted UniFrac tests. However, we did observe clear partitioning by population using the unweighted UniFrac measure, corresponding to a pattern of bacterial lineage turnover among rare members of the skin microbiome. In addition, we observed a positive correlation between individual community dissimilarity and population-level genetic differentiation. These two patterns suggest the importance of host and symbiont adaptations to varying environments (Lozupone and Knight, 2005). It is interesting to consider how associations between hellbenders and the microbiome can vary at higher taxonomic levels. Considering variation in the hellbender skin microbiome might be useful to evaluate loss of immunocompetence in the eastern hellbender’s sister subspecies, the Ozark hellbender. Within their limited range in Missouri and Arkansas, the endangered Ozark hellbender expresses a high frequency of chronic cutaneous wounds that often result in necrosis of tissues of the limbs and face (Wheeler et al., 2002; Hiler et al., 2005). A previous study evaluated the microbiome communities between the two hellbender subspecies, and characterized divergence in skin communities of both populations (Hernández-Gómez et al., 2017). However, that study only sampled one population of each subspecies; and thus, the differentiation patterns encountered could result from environmental rather than host-specific associations. Therefore, expanding characterization of the microbiome across distinct environments may be beneficial to observe whether the microbiome truly diverges between these two subspecies.

### Considerations for Hellbender Conservation

Patterns of symbiont distribution in eastern hellbender skin metacommunities can inform current conservation management. Hellbender host characteristics (e.g., immunity) and environmental variables may create a gradient of suitable habitat for cutaneous microbial symbionts. Among populations of hellbenders, we have characterized variation in symbiont dispersal and possible physiological constraints/adaptive trade-offs that result in varying patterns of taxonomic turnover (i.e., species replacements). Hellbenders have experienced population reductions due to habitat loss, water quality degradation, harvesting, and disease (Furniss et al., 2003; Nickerson and Briggler, 2007; Federal Register, 2011). Recently, hellbender conservation efforts have focused on captive breeding, captive rearing, and translocations in attempts to recover populations (Bodinof et al., 2012; Ettling et al., 2013). Captive hellbenders are currently reared in aseptic conditions that are absent of environmental sources of microbes (Ettling et al., 2013). In fact, divergent community composition has been documented between the skin microbiome of captive and wild hellbenders in Missouri (Hernández-Gómez, unpublished data) and among other amphibians (Antwis et al., 2014; Becker et al., 2014; Loudon et al., 2014). Eliminating natural sources of microbial symbionts can have consequences on the health, adaptability, and immunity of captive individuals (Redford et al., 2012).

It is important to evaluate whether captive eastern hellbenders maintain associations with the core microbes identified in this study (i.e., Proteobacteria, Actinobacteria, Firmicutes, Cyanobacteria, and Verrucomicrobia). Similar microbes are also described as core members of the skin microbiome of wild hellbenders and other amphibians (McKenzie et al., 2012; Kueneman et al., 2014; Hernández-Gómez et al., 2017), indicating important associations between these symbionts and hosts. Finding ways to assimilate the captive microbiome to naturally occurring communities in wild counterparts may increase re-introduction success. For skin communities, environmental reservoirs such as water, soil, or organic material can serve as a vehicle to introduce “wild” microbes in captivity. Both Loudon et al. (2014) and Walke et al. (2014) have described the use of environmental reservoirs (water and soil respectively) to colonize the communities on the skin of amphibians. While we did not sample all possible environmental reservoirs in the wild hellbender habitat (e.g., sediment, rocks, alternative hosts), we did find similarities between river water and the skin of hellbenders. Therefore, river water from future release sites can be used in controlled microbe exposures to alter the diversity of the skin microbiota of captive hellbenders. This technique also has the potential to be applied to captive individuals prior to release. Future investigations should focus on evaluating the effects of controlled microbial exposures in captivity, timing of assimilation of novel microbes into the skin, and how an assimilated microbiome can influence translocation success.

Conservation management can benefit by incorporating microbial barriers into translocation planning as well. Currently, translocation design is performed following recommendations from population genetics studies (Unger et al., 2013). However, we encountered barriers to microbial distributions at the genetic deme level. Translocating animals across metacommunity compartments can result in: (1) erosion of natural barriers to pathogen dispersal (Cunningham, 1996; Redford et al., 2012) and (2) alterations to the structure of the amphibian skin microbiome (dysbiosis) due to different environmental characteristics (Loudon et al., 2014). Movement of amphibians has contributed to the propagation of lethal amphibian pathogens throughout the globe (Daszak et al., 1999). In addition, the effects of exposure to numerous novel bacteria on the health of translocates is unknown. Environment-induced dysbiosis could also alter the protective phenotype of the host–microbe association resulting in increased susceptibility to pathogens (Willing et al., 2011; Woodhams et al., 2011). To limit dispersal of pathogens or exposure to novel microbes, biologists should limit heterogeneity in symbiont composition between source and supplemented populations. Therefore, conservation managers should consider symbiont boundaries in planning animal movement within each genetic deme. Increasing microbiome sampling efforts throughout each genetic deme may also increase the resolution of the boundaries that we observed.

## Author Contributions

OH-G, JH, and RW contributed in the research design. OH-G performed sample collection, laboratory work, and bioinformatics analysis. Statistical analysis was performed by OH-G with input from JH. The manuscript was written by OH-G with input from JH and RW.

## Conflict of Interest Statement

The authors declare that the research was conducted in the absence of any commercial or financial relationships that could be construed as a potential conflict of interest.

## Footnotes

1 http://qgis.osgeo.org
